# Association between hemoglobin glycation index and new-onset diabetes mellitus in individuals with chronic liver disease: Findings from a prospective cohort study using CHARLS data

**DOI:** 10.1097/MD.0000000000049567

**Published:** 2026-07-03

**Authors:** Yanyan Mao, Hui Kong, Li Wang, Fangyan Liao, Xiangwen Li

**Affiliations:** a Department of Health Management Center, Hubei Provincial Hospital of Traditional Chinese Medicine, Wuhan, China; b Department of General Surgery, Hubei Provincial Hospital of Traditional Chinese Medicine, Wuhan, China.

**Keywords:** CHARLS, chronic liver disease, diabetes mellitus, hemoglobin glycation index

## Abstract

The hemoglobin glycation index (HGI), which measures the difference between observed and predicted glycated hemoglobin, is strongly associated with diabetic complications. However, its relationship with new-onset diabetes in patients with chronic liver disease (CLD) remains unexplored. This study aimed to investigate this association using data from the China Health and Retirement Longitudinal Study database. This study included 329 participants aged ≥45 years with CLD from the China Health and Retirement Longitudinal Study database. HGI was calculated as glycated hemoglobin − (0.013 × fasting plasma glucose + 4.804). Based on baseline HGI levels, participants were categorized into 3 groups (Q1–Q3). The primary endpoint was the occurrence of new-onset diabetes mellitus events. The Kaplan–Meier curves, multivariable Cox proportional hazards models, and restricted cubic spline analysis were applied to explore the association between baseline HGI levels and the risk of diabetes mellitus incidence among individuals with CLD. In the adjusted multivariable Cox regression analysis, HGI was significantly associated with a 53% increased risk of diabetes (hazard ratio = 1.53, 95% confidence interval: 1.17–1.99, *P* = .002). The Kaplan–Meier curve analysis revealed a significant difference in the occurrence of diabetes mellitus among the HGI groups. Significant risk thresholds were identified at HGI = −0.94, beyond which diabetes mellitus risk increased substantially. Stratified and interaction analyses confirmed the stability of this association. An increase in baseline HGI levels was significantly associated with the risk of diabetes mellitus in individuals with CLD. HGI can be used as a potential indicator for predicting the long-term risk of diabetes incidence in such patients.

## 1. Introduction

Chronic liver disease (CLD) represents a significant global health burden, encompassing a spectrum of etiologies that can progress to decompensated cirrhosis, hepatocellular carcinoma, and liver-related death.^[1–5]^ Among these, the rising prevalence of nonalcoholic fatty liver disease (NAFLD) has established it as a major contributor to the CLD burden worldwide.^[6]^ Type 2 diabetes mellitus (T2DM) strongly predicts both the presence and severity of metabolic dysfunction-associated NAFLD and is a major risk factor for disease progression in other liver diseases, likely due to the synergistic interplay between hepatic steatosis/steatohepatitis and the underlying liver condition.^[7–10]^ As a metabolic disorder characterized by insulin resistance and impaired insulin secretion, T2DM exhibits a well-established bidirectional association with NAFLD, as evidenced by substantial clinical evidence.^[11,12]^ The clinical relevance of this interaction extends beyond NAFLD, as emerging evidence highlights the importance of glycemic control in patients with various CLD etiologies, where diabetes can significantly worsen prognosis.^[13]^

Glycated hemoglobin (HbA1c) serves as a biomarker reflecting glycemic control over the preceding 8 to 12 weeks in individuals with diabetes and is also utilized as a diagnostic criterion for diabetes and prediabetes.^[14,15]^ However, HbA1c levels are influenced by multiple factors, including erythrocyte lifespan. Variations in the kinetics of intraerythrocytic glycation may further lead to clinically significant underestimation or overestimation of true glycemic status.^[16]^ This implies that HbA1c may not be a reliable biomarker in specific clinical contexts. To address these limitations, the hemoglobin glycation index (HGI) has been proposed as a complementary metric to refine the interpretation of glycemic control.

The HGI quantifies interindividual variation in the relationship between HbA1c and circulating glucose concentrations.^[17]^ It is mathematically defined as the residual difference between observed HbA1c and predicted HbA1c derived from a linear regression model based on fasting plasma glucose (FPG).^[18]^ Previous studies indicate that the HGI serves as a relatively straightforward biomarker for assessing glycemic variability in patients.^[19]^ However, scant attention has been paid to glycemic variability in individuals with CLD. Investigating the correlation between HGI and incident diabetes in patients with CLD may elucidate the potential relationship between glycemic variability and diabetes pathogenesis. Furthermore, because HGI integrates both long-term glycemic control (reflected by HbA1c) and immediate glycemic status (reflected by FPG), its utility in predicting diabetes development warrants exploration. Given the critical impact of diabetes on CLD prognosis, determining whether HGI acts as a predictor of diabetes could facilitate the identification of CLD subgroups at heightened risk of adverse outcomes. The potential predictive value of HGI for diabetes has been explored in other populations,^[20,21]^ yet its role in the context of CLD remains unknown. However, CLD comprises diverse etiologies (such as NAFLD, alcoholic liver disease [ALD], and viral hepatitis) with potentially distinct pathophysiological mechanisms – such as variations in insulin resistance, inflammation, or direct viral effects^[22]^ – that may differentially affect the relationship between HGI and incident diabetes.

Notably, virtually no studies have evaluated the association between HGI and diabetes incidence in CLD populations. Accordingly, using data from the China Health and Retirement Longitudinal Study (CHARLS), this study aimed to investigate the association between HGI and the incidence of diabetes in individuals with CLD.

## 2. Methods

### 2.1. Study participants

**Figure 1.**
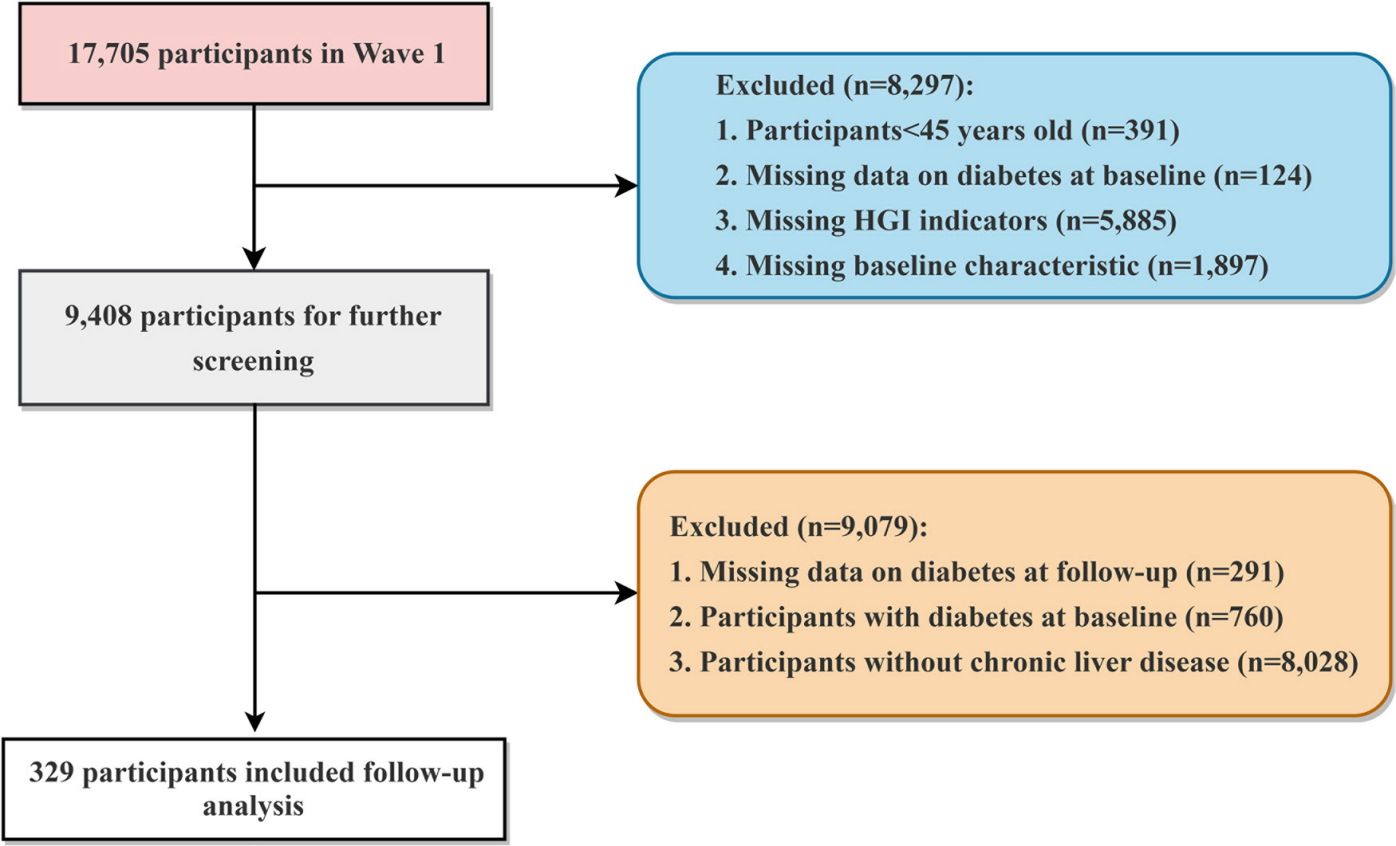
Flowchart of study participants. HGI = hemoglobin glycation index.

CHARLS, a prospective nationally representative cohort in China, utilizes a multistage, probability-proportional-to-size sampling strategy to generate longitudinal health data among community-dwelling adults aged 45 years or older. Participants underwent follow-up assessments at approximately biennial intervals (every 2–3 years) to ascertain health status. To date, 5 core survey waves have been completed, capturing longitudinal data from 2011, 2013, 2015, 2018, and 2020.^[23]^ We initially enrolled 17,705 participants in CHARLS wave 1. Among these participants, 17,376 were excluded for meeting the following exclusion criteria: missing data on HGI, FPG, glycosylated hemoglobin (HbA1c; n = 5885), age <45 years or missing data on age (n = 391), lack of data on baseline characteristics (n = 1897), missing data on stroke at baseline (n = 124), lost to follow-up (n = 291), history of diabetes (n = 760), and individuals without CLD (n = 8028). Finally, 329 participants were divided into 3 groups according to the baseline HGI quartiles and were followed up until 2020 (Fig. 1). The CHARLS study received ethical approval from the Biomedical Ethics Review Committee of Peking University (IRB00001052-11015), and all participants provided their written informed consent.

### 2.2. Exposures and outcome

**Figure 2.**
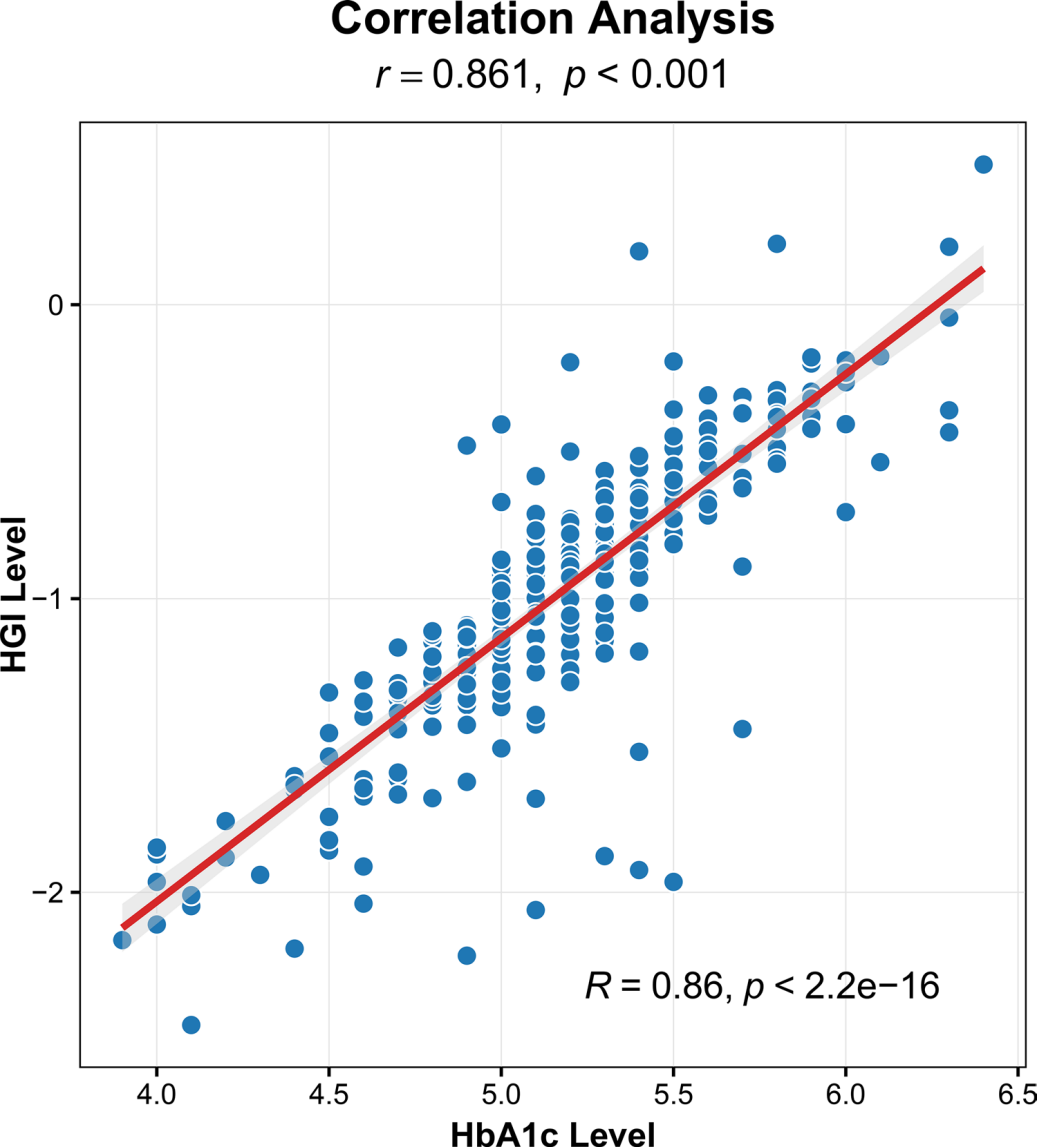
The correlation between HGI and HbA1c. HbA1c = glycated hemoglobin, HGI = hemoglobin glycation index.

The HGI was obtained by following the formula: predicted HbA1c was calculated (predicted HbA1c = 0.013 × FPG + 4.804) and the difference between the observed and predicted HbA1c values was subsequently calculated as the HGI.^[17]^ The correlation between HGI and HbA1c is shown in Figure 2. Incident diabetes was defined as the first occurrence of diabetes during follow-up, ascertained through standardized participant interviews with the question: “Has a doctor ever told you that you have diabetes?” or “Are you now taking any of the following treatments to diabetes?” The event time was the time to first diabetes diagnosis confirmed by standardized interview. To maximize statistical power while ensuring validity, we defined loss to follow-up exclusively as absence from all 4 follow-up waves (2013, 2015, 2018, and 2020).

### 2.3. Covariates

Trained interviewers collected participant data on demographics (e.g., age, sex, and marital status), health status, and functional characteristics (including smoking, alcohol consumption, hypertension, and diabetes) using standardized questionnaires. Hypertension was defined as a self-reported physician diagnosis, antihypertensive medication use, or a mean systolic blood pressure ≥140 mm Hg or diastolic blood pressure ≥90 mm Hg.^[24]^ Diabetes was defined as a self-reported physician diagnosis or current glucose-lowering therapy. Dyslipidemia, cardiovascular disease, and stroke history were ascertained through self-reported physician diagnoses. Body mass index was calculated as weight divided by height squared.

### 2.4. Statistical analysis

Continuous variables with non-normal distributions were presented as median and interquartile range, while normally distributed continuous variables were expressed as mean ± standard deviation. Categorical variables were summarized as frequencies and percentages (n, %).

For group comparisons, HGI levels were stratified into tertiles (Q1–Q3). Categorical data were analyzed using the Pearson chi-square test. Normally distributed continuous variables were compared with analysis of variance (for ≥3 groups) or Student *t* test (for 2 groups). Non-normally distributed variables were assessed using the Kruskal–Wallis test. Correlation analyses employed the Spearman rank-order method.

Cox proportional hazards models were employed to evaluate HGI as a risk factor for outcome events, with the lowest tertile (Q1) serving as the reference group. Kaplan–Meier survival analysis stratified by HGI tertiles was conducted, and between-group differences were assessed using the log-rank test. Restricted cubic splines (RCS) with 3 knots were fitted to explore the nonlinear relationship between HGI and outcome events. Threshold effect models were subsequently constructed to identify inflection points of HGI. Further subgroup analyses were performed to verify the robustness of the findings. All statistical analyses were executed in R Studio (version 4.4.2; Posit PBC), with two-sided *P*-values < .05 considered statistically significant.

## 3. Results

### 3.1. General characteristics of participants

Baseline characteristics of the study population according to HGI tertiles are summarized in Table 1. Overall, participants (N = 329) had a mean age of 59.2 ± 8.6 years, and 49.5% were women. Key metabolic parameters showed graded increases across ascending HGI tertiles. Compared with participants in the lowest HGI tertile (Q1), those in higher HGI tertiles demonstrated significantly elevated levels of FPG, HbA1c, triglycerides, low-density lipoprotein cholesterol, and body mass index. Conversely, stroke prevalence decreased progressively from Q1 to Q3 (6.4% vs 0%, *P* = .03). No other statistically significant differences in baseline characteristics were observed across tertiles.

**Table 1:** Baseline characteristics of participants according to the HGI.

Characteristics	Total	HGI			*P*-value
		Q1	Q2	Q3	
N	329	110	109	110
Sex (%)					.825
Male	166 (50.5)	56 (50.9)	57 (52.3)	53 (48.2)	
Female	163 (49.5)	54 (49.1)	52 (47.7)	57 (51.8)	
Age, yr (mean [SD])	59.2 (8.6)	57.9 (8.4)	60.1 (8.6)	59.6 (8.9)	.147
Education level (%)					.366
High school+	32 (9.7)	10 (9.1)	14 (12.8)	8 (7.3)	
Primary/illiterate	297 (90.3)	100 (90.9)	95 (87.2)	102 (92.7)	
Marital status (%)					.479
Married	283 (86.0)	94 (85.5)	91 (83.5)	98 (89.1)	
Others	46 (14.0)	16 (14.5)	18 (16.5)	12 (10.9)	
Location (%)					.809
City	19 (5.8)	7 (6.4)	5 (4.6)	7 (6.4)	
Village	310 (94.2)	103 (93.6)	104 (95.4)	103 (93.6)	
Smoking status (%)					.523
Yes	140 (42.6)	42 (38.2)	49 (45.0)	49 (44.5)	
No	189 (57.4)	68 (61.8)	60 (55.0)	61 (55.5)	
Drink (%)					.066
Yes	75 (22.8)	31 (28.2)	27 (24.8)	17 (15.5)	
No	254 (77.2)	79 (71.8)	82 (75.2)	93 (84.5)	
BMI, kg/m^2^ (mean [SD])	23.4 (3.7)	23.4 (3.5)	22.7 (3.3)	24.1 (4.2)	.029
Hypertension (%)					.101
Yes	135 (41.0)	53 (48.2)	37 (33.9)	45 (40.9)	
No	194 (59.0)	57 (51.8)	72 (66.1)	65 (59.1)	
Stroke (%)					.031
Yes	11 (3.3)	7 (6.4)	4 (3.7)	0 (0.0)	
No	318 (96.7)	103 (93.6)	105 (96.3)	110 (100.0)	
Dyslipidemia (%)					.066
Yes	137 (41.6)	48 (43.6)	36 (33.0)	53 (48.2)	
No	192 (58.4)	62 (56.4)	73 (67.0)	57 (51.8)	
Heart problems (%)					.227
Yes	64 (19.5)	20 (18.2)	17 (15.6)	27 (24.5)	
No	265 (80.5)	90 (81.8)	92 (84.4)	83 (75.5)	
FPG, mg/dL (mean [SD])	103.6 (19.2)	111.2 (23.9)	100.6 (10.5)	99.1 (18.4)	<.001
HbA1c, mg/dL (mean [SD])	5.2 (0.4)	4.8 (0.3)	5.2 (0.2)	5.6 (0.3)	<.001
TG, mg/dL (mean [SD])	137.5 (124.0)	168.4 (180.5)	108.9 (72.1)	134.9 (82.3)	.002
HDL, mg/dL (mean [SD])	50.3 (15.1)	47.8 (16.0)	52.6 (13.5)	50.6 (15.4)	.059
LDL, mg/dL (mean [SD])	111.0 (34.2)	104.7 (37.9)	111.8 (32.3)	116.5 (31.4)	.036
TC, mg/dL (mean [SD])	189.0 (37.6)	187.9 (42.5)	185.5 (34.2)	193.4 (35.6)	.283

BMI = body mass index, FPG = fasting plasma glucose, HbA1c = glycated hemoglobin, HGI = hemoglobin glycation index, LDL = low-density lipoprotein, SD = standard deviation, TG = triglycerides.

### 3.2. The correlation between HGI and HbA1c

Our analysis demonstrated a remarkably strong positive correlation between HGI and HbA1c levels (*r* = 0.861, *P* < .001; Fig. 2). This highly significant association indicates that HGI, as a calculated measure of glycemic variability, captures a substantial proportion of the physiological variation reflected in HbA1c, and emerges as a crucial, complementary tool for a more holistic evaluation of long-term glycemic management.

### 3.3. Association of HGI with the risk of new-onset diabetes

**Figure 3.**
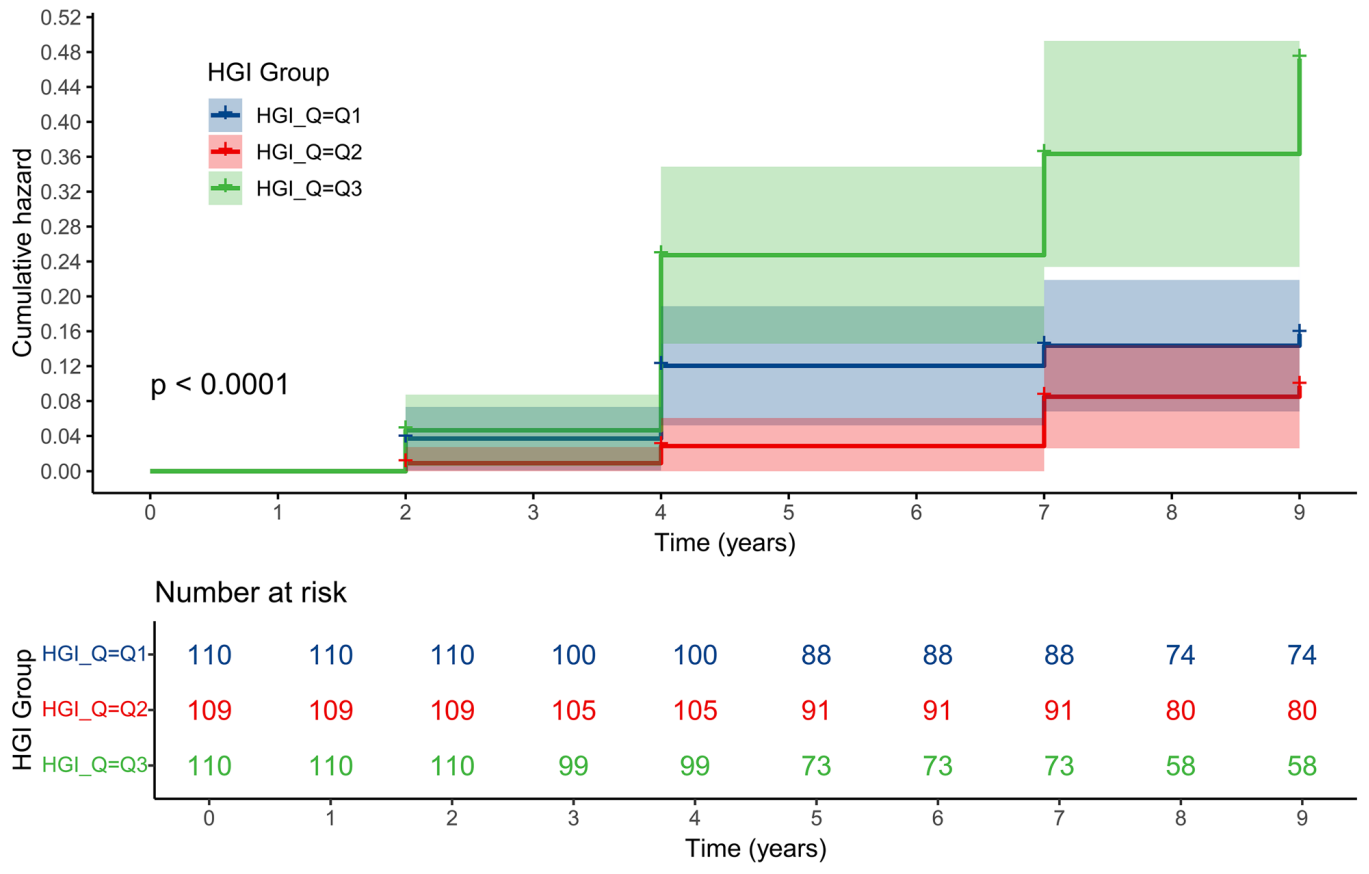
Kaplan–Meier curves for diabetes incidence stratified by HGI value. HGI = hemoglobin glycation index.

As depicted in Figure 3, during a mean follow-up of 8.72 years, diabetes incidence rates across HGI tertiles were 13.64% (Q1 group), 9.00% (Q2 group), and 33.63% (Q3 group). Kaplan–Meier analysis revealed a statistically significant divergence in cumulative diabetes incidence between the lowest (Q1) and highest (Q3) HGI tertiles (log-rank *P* < .001).

Cox proportional hazards models (Table 2) demonstrated that elevated baseline HGI was significantly associated with increased diabetes risk. When evaluated as both continuous and categorical variables, in the unadjusted Model 1, HGI showed a significant association with diabetes risk. Each 1-unit increment in HGI corresponded to a hazard ratio (HR) of 1.64 (95% confidence interval [CI]: 1.26–2.13), representing a 64% increased risk. After comprehensive adjustment for potential confounders in Model 3, baseline HGI remained significantly associated with diabetes risk (HR = 1.53, 95% CI: 1.17–1.99). Additionally, in the fully adjusted Model 3, the highest HGI tertile (Q3) exhibited significantly increased diabetes risk compared to the lowest tertile (Q1; HR = 2.77, 95% CI: 1.46–5.26; *P *= .002).

**Table 2:** The relationship of HGI to diabetes incidence.

	Case	Model 1 (HR, 95% CI)	*P*-value	Model 2 (HR, 95% CI)	*P*-value	Model 3 (HR, 95% CI)	*P*-value
HGI (per SD)		1.64 (1.26–2.13)	<.001	1.52 (1.16–1.98)	.002	1.53 (1.17–1.99)	.002
HGI quartile							
Q1	15 (13.64%)	Ref		Ref		Ref	
Q2	9 (8.26%)	0.57 (0.25–1.30)	.182	0.55 (0.24–1.26)	.157	0.64 (0.28–1.50)	.309
Q3	37 (33.63%)	2.79 (1.53–5.10)	<.001	2.46 (1.33–4.54)	.004	2.77 (1.46–5.26)	.002

Model 1: unadjusted.

Model 2: adjusted for age, sex, marital status, education levels, drinking status, smoking status, residence, and BMI.

Model 3: adjusted for factors in Model 2 and history of hypertension, dyslipidemia, stroke, and heart problems.

BMI = body mass index, CI = confidence interval, HGI = hemoglobin glycation index, HR = hazard ratio, SD = standard deviation.

### 3.4. The RCS analysis of HGI with diabetes risk

**Figure 4.**
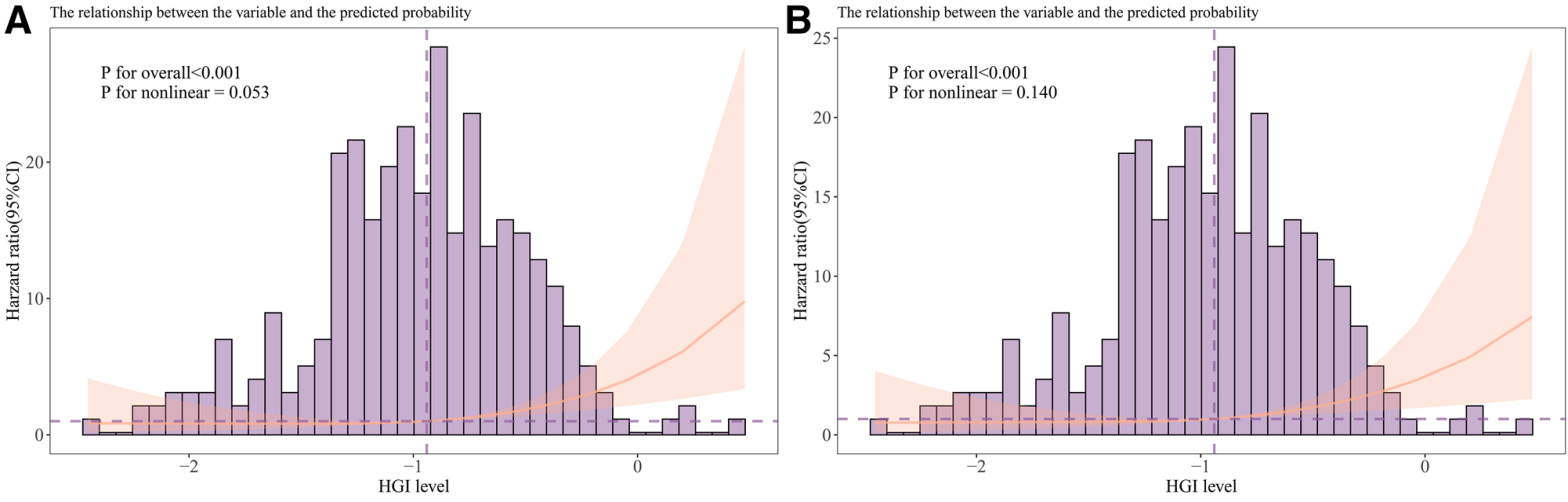
RCS analysis of HGI with diabetes incidence. (A) Unadjusted model (without controlling for covariates). (B) Multivariable-adjusted model (adjusted for age, sex, marital status, education level, drinking status, smoking status, residence, BMI, history of hypertension, dyslipidemia, stroke, and heart problems). BMI = body mass index, CI = confidence interval, HGI = hemoglobin glycation index, RCS = restricted cubic splines.

RCS analysis revealed a significant positive association between elevated baseline HGI and diabetes risk in patients with CLD. The relationship exhibited a J-shaped curve (Fig. 4A), with diabetes HRs increasing progressively beyond an inflection point. This J-shaped curve persisted robustly after multivariable adjustment for confounders. The thresholds of elevated risk were identified at HGI = −0.94, indicating potential biological thresholds for diabetes risk stratification (Fig. 4B).

### 3.5. Subgroup and interaction analysis

Subgroup analyses confirmed uniformly elevated diabetes risk associated with high HGI across all demographic and clinical strata (Table 3). Formal interaction tests detected no significant effect modification by age, sex, marital status, or history of hypertension (all *P*-interaction > .05), supporting the invariant nature of this association. Meanwhile, the significantly elevated risk of diabetes incidence observed in the Q3 group compared with the Q2 reference group was consistent across all prespecified subgroups, including age, sex, marital status, smoking status, and comorbid disease status. This robustness further strengthens the primary finding of a J-shaped association. The upward trajectory of risk beyond an HGI threshold of −0.94, as identified by our model, is precisely mirrored in this stepwise increase in risk from the Q2 group to the Q3 group.

**Table 3:** Subgroup analysis of the HGI with diabetes incidence.

Subgroup	Case/total	Q1 group	Q2 group	Q3 group	*P*-value	*P* for interaction
Sex						.98
Male	28/166	Ref	0.73 (0.20–2.62)	3.01 (1.09–8.30)	.001	
Female	33/163	Ref	0.65 (0.21–2.04)	2.67 (1.15–6.21)	.111	
Age (yr)						.32
≥60	35/154	Ref	0.87 (0.29–2.57)	2.39 (0.99–5.73)	.003	
<60	26/175	Ref	0.38 (0.07–1.91)	3.93 (1.49–10.35)	.180	
Marital status						.63
Married	54/283	Ref	0.80 (0.32–2.00)	3.31 (1.63–6.71)	<.001	
Others	7/46	Ref	0.10 (0.00–3.21)	1.46 (0.26–8.37)	.95	
Smoking status						.52
Yes	22/140	Ref	0.58 (0.10–3.23)	5.04 (1.48–17.12)	.056	
No	39/189	Ref	0.86 (0.32–2.31)	3.02 (1.34–6.79)	.016	
Hypertension						.10
Yes	34/135	Ref	0.86 (0.32–2.36)	2.81 (1.19–6.62)	.027	
No	27/194	Ref	0.31 (0.06–1.66)	3.90 (1.41–10.77)	.006	
Dyslipidemia						.23
Yes	33/137	Ref	0.71 (0.22–2.25)	1.86 (0.79–4.40)	.049	
No	28/192	Ref	0.66 (0.17–2.51)	4.73 (1.69–13.25)	.009	
Heart problems						.36
Yes	19/64	Ref	2.28 (0.40–12.98)	4.47 (1.13–17.75)	.158	
No	42/265	Ref	0.44 (0.15–1.27)	2.73 (1.30–5.70)	.012	

HGI = hemoglobin glycation index.

## 4. Discussion

Employing a comprehensive array of analytical techniques, this study investigated the association between HGI and the incidence of diabetes mellitus within a cohort of patients with CLD. Analysis of health examination data from 329 participants revealed a significant J-shaped relationship between HGI levels and diabetes risk. This association was consistently observed across subsequent subgroup analyses, substantiating the robustness of our primary findings. The present study provides novel evidence indicating that HGI, as a measure of glycemic variability, is a potential predictor of incident diabetes among individuals with CLD. To the best of our knowledge, no prior study has specifically assessed or established the predictive capacity of HGI for diabetes incidence in this high-risk population.

Given that NAFLD represents the predominant etiology of CLD globally, a substantial proportion of our CLD cohort likely comprises individuals with NAFLD. NAFLD and its advanced form, nonalcoholic steatohepatitis, signify progressive liver damage and carry a significant risk of progression to cirrhosis and hepatocellular carcinoma.^[6]^ A bidirectional relationship between NAFLD and T2DM has been extensively documented.^[25,26]^ T2DM promotes the progression of NAFLD to cirrhosis and confers a 2- to 3-fold increased risk of both liver-related and all-cause mortality.^[27,28]^ Highlighting the impact of glycemia on fibrosis progression, a recent biopsy-confirmed study involving 713 participants with NAFLD (48% with T2DM) demonstrated that each 1% increase in mean HbA1c levels was independently associated with a 15% higher odds of liver fibrosis stages.^[29]^ In addition to mean HbA1c, longitudinal HbA1c variability emerged as an independent predictor of NAFLD development, even after adjustment for established metabolic risk factors.^[30]^ In parallel, the presence of NAFLD adversely impacts both the incidence of T2DM and associated adverse clinical outcomes.^[31]^ We acknowledge that CLD encompasses etiologies beyond NAFLD, including ALD and chronic viral hepatitis. In ALD, alcohol metabolism generates oxidative stress and directly impairs pancreatic β-cell function, potentially altering the relationship between glycemic variability and incident diabetes.^[8]^ In chronic hepatitis, viral proteins can interfere with insulin signaling, leading to insulin resistance even in the absence of significant steatosis.^[32]^ These distinct pathways suggest that the predictive value of HGI for diabetes may vary across CLD subtypes. Unfortunately, the CHARLS dataset does not allow reliable etiological subclassification of CLD. Therefore, although our primary analysis was conducted in the overall CLD population, the findings should be interpreted as most applicable to NAFLD-predominant settings. Future studies with etiologically stratified designs are needed to determine whether the HGI-diabetes association holds uniformly across all CLD subtypes.

The HGI quantifies interindividual variation in the relationship between HbA1c and measured blood glucose concentrations.^[17]^ HGI has demonstrated predictive capacity for disease severity and mortality in diabetes mellitus, hypertension, coronary artery disease, and chronic kidney disease.^[33–37]^ Collectively, this evidence establishes HGI as a robust prognostic biomarker for disease progression and mortality risk across diverse populations. Meanwhile, previous studies have indicated that HGI provides a clinically accessible measure of glycemic variability.^[19]^ Beyond reflecting long-term glycemic control, HGI also correlates with immediate FPG levels. Enhanced HGI management may improve short-term glycemic outcomes. Importantly, HGI assessment serves as an effective predictor of diabetes risk in CLD patients and may facilitate identification of high-risk subpopulations.

As the foundational pathophysiological driver of diabetes, insulin resistance initiates disease progression. Compelling studies demonstrate that excessive glycemic variability accelerates β-cell dysfunction through glucotoxicity and oxidative stress, ultimately resulting in irreversible secretory failure.^[38]^ In rats, either continuous or intermittent hyperglycemia induced β-cell dysfunction and insulin resistance^[39]^; chronic oscillating glucose caused β-cell dedifferentiation and failure.^[40]^ Concurrently, the induction of oxidative stress and inflammatory responses by glycemic variability initiates a vicious cycle. The dysregulation of hepatic glucose homeostasis in CLD – characterized by impaired glycogen storage and pathological gluconeogenesis – amplifies postprandial glycemic volatility. Such persistent dysglycemia independently drives β-cell decompensation through glucolipotoxicity while aggravating insulin resistance via inflammation-mediated serine phosphorylation of insulin receptor substrates.^[6]^ In summary, increased glycemic variability could be indicative of underlying β-cell functional impairment. Therefore, HGI, as a metric quantifying glycemic variability, might act as a surrogate marker reflecting the activity of this pathophysiological cascade, ultimately underpinning its utility in predicting incident diabetes. However, the extent to which this glucotoxicity-oxidative stress cascade operates in non-NAFLD CLD remains less clear. Such heterogeneity warrants cautious extrapolation of our mechanistic interpretation to the entire CLD spectrum.

Several key limitations warrant consideration: the observed HGI-diabetes association cannot establish causality due to the inherent constraints of observational designs; unmeasured confounders may persist despite extensive covariate adjustment; the absence of data on the etiological spectrum of CLD limits our ability to assess whether the HGI-diabetes association varies across different CLD subtypes; diabetes mellitus was identified based on self-reported physician diagnosis, FPG, and HbA1c criteria; however, reliance on self-report may introduce recall bias and potential misclassification, particularly in underdiagnosed or asymptomatic cases; heterogeneous follow-up schedules and lag between biochemical onset and clinical diagnosis introduce temporal misclassification in incidence estimates; and cohort restrictions to CLD patients yielded reduced statistical power post-exclusions, with possible selection bias. Prospective validation in multi-ethnic cohorts with protocol-driven follow-up, incorporating detailed etiological characterization and standardized diabetes ascertainment, is essential to confirm these findings and advance clinical implementation.

## 5. Conclusion

In conclusion, this prospective cohort study suggests that a higher HGI is independently associated with an increased risk of new-onset diabetes among individuals with CLD. Moreover, due to the etiological heterogeneity of CLD and the lack of subtype classification in the current dataset, future studies are warranted to validate our results in NAFLD, ALD, and viral hepatitis separately.

## Acknowledgments

We extend our sincere gratitude to the CHARLS team for their invaluable efforts and to all the respondents whose data were instrumental in this study.

## Author contributions

**Conceptualization:** Yanyan Mao, Xiangwen Li.

**Data curation:** Yanyan Mao.

**Formal analysis:** Yanyan Mao, Fangyan Liao.

**Methodology:** Yanyan Mao, Hui Kong, Xiangwen Li.

**Validation:** Hui Kong, Li Wang.

**Visualization:** Hui Kong, Li Wang.

**Supervision:** Fangyan Liao, Xiangwen Li.

**Project administration:** Xiangwen Li.

**Writing – original draft:** Yanyan Mao, Li Wang, Xiangwen Li.

**Writing – review & editing:** Yanyan Mao, Hui Kong, Fangyan Liao, Xiangwen Li.

## References

[1] GBD 2017 Cirrhosis Collaborators. The global, regional, and national burden of cirrhosis by cause in 195 countries and territories, 1990–2017: a systematic analysis for the Global Burden of Disease Study 2017. Lancet Gastroenterol Hepatol. 2020;5:245–66. 31981519 10.1016/S2468-1253(19)30349-8 PMC7026710

[2] Danjuma MI Naseralallah L Ansari S. Prevalence and global trends of polypharmacy in patients with chronic liver disease: a systematic review and meta-analysis. Medicine (Baltimore). 2023;102:e32608. 37171329 10.1097/MD.0000000000032608 PMC10174406

[3] Le Couteur DG Ngu MC Hunt NJ Brandon AE Simpson SJ Cogger VC. Liver, ageing and disease. Nat Rev Gastroenterol Hepatol. 2025;22:680–95. 40721658 10.1038/s41575-025-01099-z

[4] Satapathy SK Sundaram V Shiffman ML Jamieson BD. Real-world use of avatrombopag in patients with chronic liver disease and thrombocytopenia undergoing a procedure. Medicine (Baltimore). 2023;102:e35208. 37800793 10.1097/MD.0000000000035208 PMC10553023

[5] Zhao X Xu D Ji W. Global and Chinese burden of non-alcoholic fatty liver disease in chronic liver disease: findings from the Global Burden of Disease Study 2021. Chin Med J (Engl). 2025;138:1741–51. 40539304 10.1097/CM9.0000000000003726 PMC12273651

[6] Loomba R Friedman SL Shulman GI. Mechanisms and disease consequences of nonalcoholic fatty liver disease. Cell. 2021;184:2537–64. 33989548 10.1016/j.cell.2021.04.015 PMC12168897

[7] Ajmera V Cepin S Tesfai K. A prospective study on the prevalence of NAFLD, advanced fibrosis, cirrhosis and hepatocellular carcinoma in people with type 2 diabetes. J Hepatol. 2023;78:471–8. 36410554 10.1016/j.jhep.2022.11.010 PMC9975077

[8] Cusi K Abdelmalek MF Apovian CM. Metabolic dysfunction-associated steatotic liver disease (MASLD) in people with diabetes: the need for screening and early intervention. A consensus report of the American Diabetes Association. Diabetes Care. 2025;48:1057–82. 40434108 10.2337/dci24-0094

[9] Qi X Li J Caussy C Teng GJ Loomba R. Epidemiology, screening, and co-management of type 2 diabetes mellitus and metabolic dysfunction-associated steatotic liver disease. Hepatology. 2026;83:661–78. 38722246 10.1097/HEP.0000000000000913 PMC12904236

[10] Steinberg GR Valvano CM De Nardo W Watt MJ. Integrative metabolism in MASLD and MASH: pathophysiology and emerging mechanisms. J Hepatol. 2025;83:584–95. 40032040 10.1016/j.jhep.2025.02.033

[11] Du M Yang H Niu J. Umbilical cord-mesenchymal stromal cell-derived extracellular vesicles target the liver to improve neurovascular health in type 2 diabetes with non-alcoholic fatty liver disease. J Extracell Vesicles. 2025;14:e70125. 40620065 10.1002/jev2.70125 PMC12230362

[12] Mao X Zhang X Lai R. Glucagon-like peptide 1 receptor agonist and reduced liver and non-liver complications in adults with type 2 diabetes and metabolic dysfunction-associated steatotic liver disease: a target trial emulation study. Clin Mol Hepatol. 2025;31:1084–99. 40268291 10.3350/cmh.2024.1096 PMC12260620

[13] Xing Y Zhen Y Yang L Huo L Ma H. Association between hemoglobin glycation index and non-alcoholic fatty liver disease. Front Endocrinol (Lausanne). 2023;14:1094101. 36824362 10.3389/fendo.2023.1094101 PMC9941148

[14] Vilsbøll T Malecki MT Sharma P Thieu VT Chivukula KK Kiljanski J. HbA1c reduction with tirzepatide in people with type 2 diabetes: the contribution of weight loss assessed by a mediation analysis. Diabetes Obes Metab. 2025;27:5498–505. 40746012 10.1111/dom.16592 PMC12409195

[15] Su Y Xia C Zhang H. Emerging biosensor probes for glycated hemoglobin (HbA1c) detection. Mikrochim Acta. 2024;191:300. 38709399 10.1007/s00604-024-06380-7

[16] Wang Z Guo H Teng X. Diabetic retinopathy and mortality in adults with diabetes: causal mediation analysis of the role of HbA1c. Diabetes Res Clin Pract. 2025;229:112936. 41075986 10.1016/j.diabres.2025.112936

[17] Zhou W Zhang L Liu T. Association between the hemoglobin glycation index (HGI) and risk of diabetic nephropathy: a retrospective cohort study. Diabetes Metab Syndr Obes. 2025;18:1859–72. 40491573 10.2147/DMSO.S523442 PMC12147926

[18] Klein KR Franek E Marso S. Hemoglobin glycation index, calculated from a single fasting glucose value, as a prediction tool for severe hypoglycemia and major adverse cardiovascular events in DEVOTE. BMJ Open Diabetes Res Care. 2021;9:e002339. 10.1136/bmjdrc-2021-002339 PMC8614152 34819298

[19] Lyu L Yu J Liu Y. High hemoglobin glycation index is associated with telomere attrition independent of HbA1c, mediated by TNFα. J Clin Endocrinol Metab. 2022;107:462–73. 34562085 10.1210/clinem/dgab703

[20] Wang M Li S Zhang X Li X Cui J. Association between hemoglobin glycation index and non-alcoholic fatty liver disease in the patients with type 2 diabetes mellitus. J Diabetes Investig. 2023;14:1303–11. 10.1111/jdi.14066 PMC10583654 37551797

[21] Wu QY Mo LR Nan J Huang WZ Wu Q Su Q. The association between the hemoglobin glycation index and cardiometabolic diseases: a mini-review. J Clin Hypertens (Greenwich). 2025;27:e70092. 40662995 10.1111/jch.70092 PMC12261972

[22] Ho GJK Tan FXN Sasikumar NA. High global prevalence of steatotic liver disease and associated subtypes: a meta-analysis. Clin Gastroenterol Hepatol. 2025;23:2423–32.e1. 40204206 10.1016/j.cgh.2025.02.006

[23] Shao X Yu T Luo L Zheng H Yang F Dong J. LDL-C combined with Chinese visceral adiposity index as a risk stratification tool for cardiometabolic multimorbidity in middle-aged and older Chinese adults: a national prospective cohort study. Cardiovasc Diabetol. 2026;25:176. 42036685 10.1186/s12933-026-03195-z PMC13274176

[24] Williams B Mancia G Spiering W. 2018 ESC/ESH guidelines for the management of arterial hypertension. Eur Heart J. 2018;39:3021–104. 30165516 10.1093/eurheartj/ehy339

[25] Wang X Zhao D Cheng L Gao J Li J Geng C. Mendelian randomization explores the causal relationships between obesity, diabetes, inflammation and nonalcoholic fatty liver disease. Medicine (Baltimore). 2023;102:e34638. 37747017 10.1097/MD.0000000000034638 PMC10519523

[26] Chaudhary S Manocha K Malik P Aggarwal M Rao R Garg M. Diabetes and NAFLD: a synergistic threat to metabolic health. Adv Pharm Bull. 2025;15:766–78. 41835049 10.34172/apb.025.44094 PMC12980267

[27] Jing M Jiang Y. Microbiome-mediated crosstalk between T2DM and MASLD: a translational review focused on function. Front Endocrinol (Lausanne). 2025;16:1677175. 41334445 10.3389/fendo.2025.1677175 PMC12665569

[28] Zhang J Li J Zheng Y. Lipid metabolism and lipid signaling in extracellular vesicles ontogeny: from biogenesis to functional execution. J Nanobiotechnol. 2025;23:672. 10.1186/s12951-025-03769-1 PMC12519735 41084000

[29] Alexopoulos A-S Crowley MJ Wang Y. Glycemic control predicts severity of hepatocyte ballooning and hepatic fibrosis in nonalcoholic fatty liver disease. Hepatology. 2021;74:1220–33. 33724511 10.1002/hep.31806 PMC8518519

[30] Yoo JH Kang M Kim G. Mean and visit-to-visit variability of glycated hemoglobin, and the risk of non-alcoholic fatty liver disease. J Diabetes Investig. 2021;12:1252–62. 10.1111/jdi.13455 PMC8264391 33135331

[31] Mantovani A Petracca G Beatrice G Tilg H Byrne CD Targher G. Non-alcoholic fatty liver disease and risk of incident diabetes mellitus: an updated meta-analysis of 501 022 adult individuals. Gut. 2021;70:962–9. 32938692 10.1136/gutjnl-2020-322572

[32] Hu JH Chang ML Lin MS Huang TJ Hsieh YY. Effect of direct-acting antiviral therapy on glycemic control in patients with chronic hepatitis C and type 2 diabetes: a systematic review and meta-analysis. Viruses. 2026;18:239. 41754582 10.3390/v18020239 PMC12945286

[33] Ibarra-Salce R Pozos-Varela FJ Martinez-Zavala N. Correlation between hemoglobin glycation index measured by continuous glucose monitoring with complications in type 1 diabetes. Endocr Pract. 2023;29:162–7. 36627022 10.1016/j.eprac.2023.01.001

[34] Wu J Liang D-L Xie Y. Association between hemoglobin glycation index and risk of cardiovascular disease and all-cause mortality in type 2 diabetic patients: a meta-analysis. Front Cardiovasc Med. 2021;8:690689. 34124211 10.3389/fcvm.2021.690689 PMC8193090

[35] Wei X Chen X Zhang Z. Risk analysis of the association between different hemoglobin glycation index and poor prognosis in critical patients with coronary heart disease-a study based on the MIMIC-IV database. Cardiovasc Diabetol. 2024;23:113. 38555454 10.1186/s12933-024-02206-1 PMC10981833

[36] Cheng M-D Tang J-N Liu Z-Y. Association of hemoglobin glycation index with prognosis of coronary artery disease after percutaneous coronary intervention: a retrospective cohort study. Diab Vasc Dis Res. 2023;20:14791641231193306. 37561132 10.1177/14791641231193306 PMC10416663

[37] Lin L Wang AP Dou JT. Predictive value of hemoglobin glycation index for chronic kidney disease. Zhonghua Nei Ke Za Zhi. 2022;61:1310–7. 36456510 10.3760/cma.j.cn112138-20220508-00347

[38] Accili D Deng Z Liu Q. Insulin resistance in type 2 diabetes mellitus. Nat Rev Endocrinol. 2025;21:413–26. 40247011 10.1038/s41574-025-01114-y

[39] Klimontov VV Saik OV Korbut AI. Glucose variability: how does it work? Int J Mol Sci. 2021;22:7783. 34360550 10.3390/ijms22157783 PMC8346105

[40] Iida H Watada H. Pancreatic β-cell dysfunction and diabetes. Juntendo Med J. 2025;71:158–65. 40666490 10.14789/ejmj.JMJ25-0001-R PMC12257219

